# Probable Isolated Rectal Tuberculosis Presenting as Mild Chronic Proctitis in an Immunocompetent Adolescent: A Diagnostic Pitfall and Mini-Review of the Literature

**DOI:** 10.14309/crj.0000000000002297

**Published:** 2026-09-07

**Authors:** Gourab Bhaduri, Bijan Basak, Bijit Saha, Asif Ali Ahmed, Brindaa Bandopadhyay

**Affiliations:** 1 Department of Gastroenterology, The Mission Hospital, Durgapur, West Bengal, India; 2 Department of Pathology, The Mission Hospital, Durgapur, West Bengal, India; 3 Department of Surgical Gastroenterology, The Mission Hospital, Durgapur, West Bengal, India

**Keywords:** rectal tuberculosis, proctitis, granulomas

## Abstract

Rectal involvement in gastrointestinal tuberculosis (TB) is uncommon and may mimic inflammatory bowel disease or nonspecific proctitis, leading to diagnostic uncertainty. We report the case of an 18-year-old immunocompetent man presenting with chronic increased stool frequency and mucus passage, occasionally blood-streaked, without constitutional symptoms. Colonoscopy revealed nonspecific proctitis. Histopathological examination demonstrated confluent granulomas with Langhans giant cells. Microbiological confirmation including GeneXpert was negative. In view of the strongly suggestive histopathological findings and the endemic setting, a diagnosis of probable isolated TB was made, and anti-tubercular therapy was initiated. Probable rectal TB should be considered in the differential diagnosis of chronic proctitis, even in the absence of systemic features. Histopathology remains crucial, particularly in endemic regions where microbiological tests may be negative.

## INTRODUCTION

Tuberculosis (TB) remains endemic in India. Gastrointestinal TB accounts for a small proportion of extrapulmonary TB and most commonly involves the ileocecal region. Rectal involvement is rare. Davis^1^ first reported rectal TB in 1957. Despite advances in imaging and endoscopy, diagnosis remains challenging due to nonspecific clinical and endoscopic findings. We report a case of probable isolated rectal TB in an immunocompetent adolescent presenting with mild rectal symptoms.

## CASE REPORT

An 18-year-old man presented with a history of increased stool frequency for 6 to 7 months. He also reported passage of mucus in stool, occasionally blood-streaked. He denied fever, weight loss, or anorexia. He experienced intermittent spasmodic abdominal pain.

Routine biochemical parameters were within normal limits. Fecal calprotectin was marginally elevated. Sigmoidoscopy performed elsewhere showed patchy mucosal erythema in the rectum. Computed tomography (CT) enterography revealed no significant abnormalities. He was initially treated with mebeverine and topical mesalamine without symptomatic improvement.

**Figure 1.**
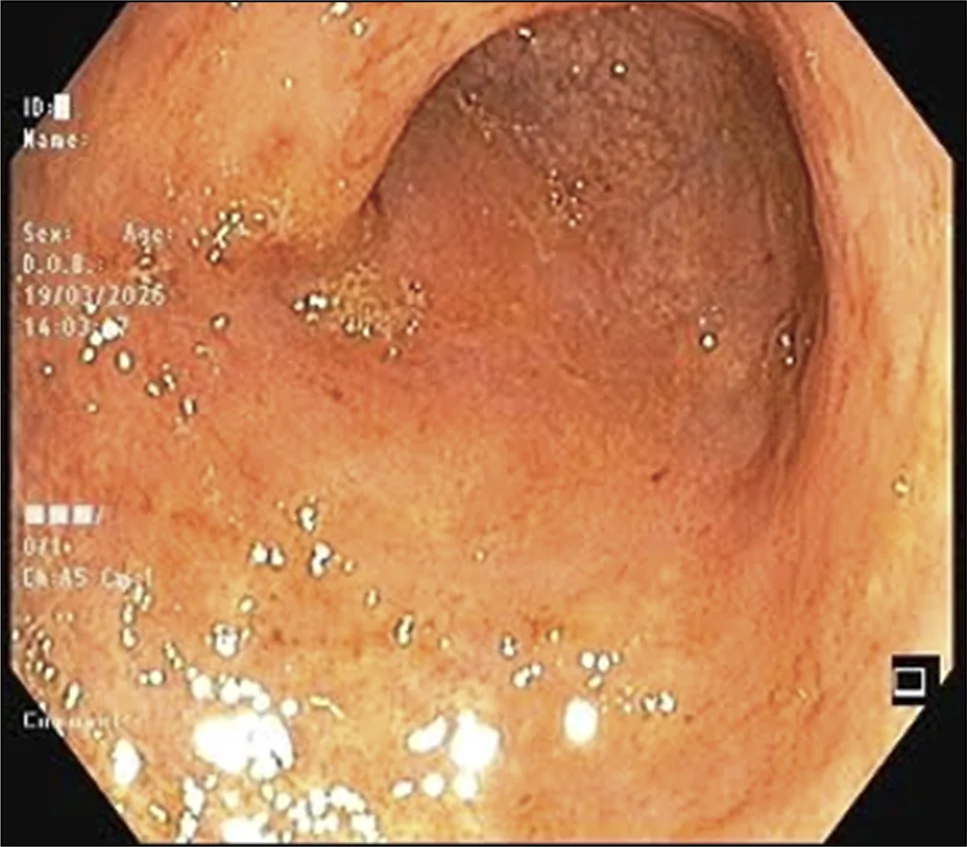
Colonoscopy showing patchy loss of vascular pattern with erosions (arrows).

**Figure 2.**
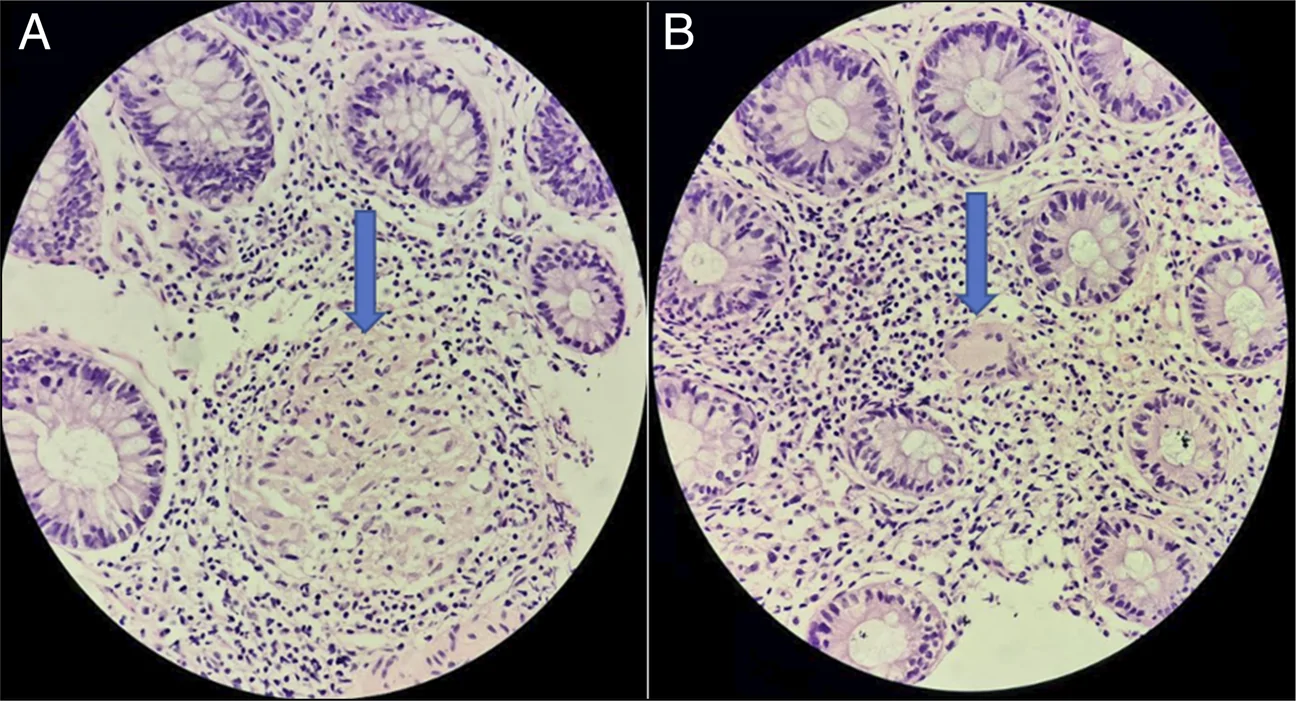
Hematoxylin and eosin stain (400×) showing (A) confluent epithelioid granuloma and (B) Langhan giant cells (arrowheads).

A full-length colonoscopy revealed patchy loss of vascular pattern with superficial erosions confined to the rectum, suggestive of nonspecific proctitis (Figure 1). Histopathological examination of rectal biopsies demonstrated multiple confluent deep seated epithelioid granulomas with Langhans giant cells (Figure 2). No caseation necrosis was identified, and Ziehl–Neelsen staining did not demonstrate acid-fast bacilli. Crypt architecture was preserved without crypt abscesses or basal plasmacytosis and ulceration with fissures.

Given the strong histopathological suspicion of TB, repeat biopsies were obtained for nucleic acid amplification testing (GeneXpert), which yielded negative results. Chest high-resolution CT did not reveal any pulmonary focus.

**Figure 3.**
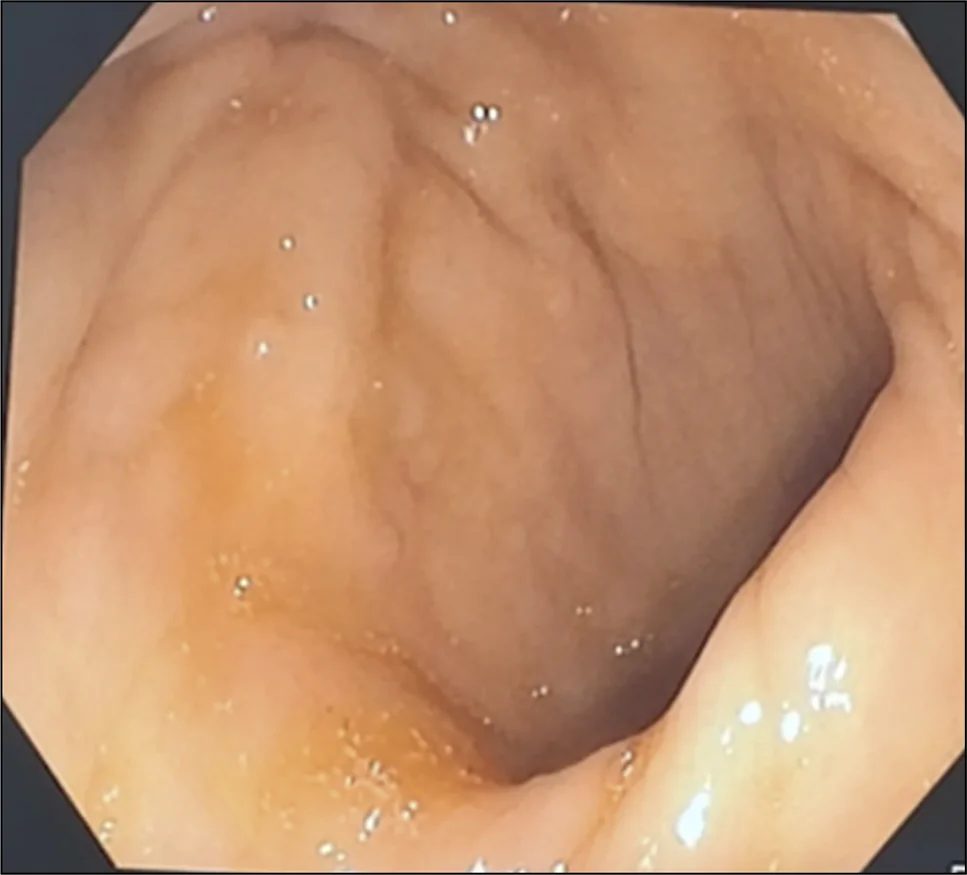
Rectal mucosal healing after 12 weeks of anti-tubercular therapy.

In view of the histological findings in an endemic setting and lack of alternative diagnosis, a diagnosis of probable isolated rectal TB was made. Following counseling, anti-tubercular therapy was initiated. He received isoniazid 300 mg, rifampicin 450 mg, ethambutol 800 mg, and pyrazinamide 1,500 mg. After 6 weeks of treatment, his symptoms, including urgency, tenesmus, and rectal bleeding, resolved completely. Sigmoidoscopy repeated after 12 weeks revealed mucosal healing (Figure 3).

## DISCUSSION

Rectal TB is an uncommon manifestation of gastrointestinal TB, accounting for a small fraction of cases even in endemic regions. The ileocecal region remains the most frequently involved site, while isolated rectal involvement is distinctly rare. Despite advances in endoscopy and molecular diagnostics, rectal TB continues to pose a diagnostic challenge due to its variable and often nonspecific clinical and endoscopic presentation.

Proposed mechanisms of rectal involvement include hematogenous dissemination, lymphatic spread from perirectal lymph nodes, or direct extension from adjacent structures.^2,3^ However, in most cases, the exact route remains speculative. The clinical presentation is often nonspecific and may include altered bowel habits, abdominal pain, hematochezia, obstruction, fistula formation, or constipation.^4,5^ Endoscopic findings are variable and may include ulceration, nodularity, strictures, or nonspecific mucosal inflammation.^6^

In the present case, the clinical presentation was limited to chronic altered bowel habits with mucus discharge and intermittent blood-streaking, without systemic symptoms such as fever, weight loss, or anorexia. Notably, colonoscopic findings were subtle, showing only patchy loss of vascular pattern with superficial erosions confined to the rectum, suggestive of nonspecific proctitis. The absence of ulceration, a mass lesion, stricture, or skip lesions contributed to a low initial index of suspicion for TB, thereby simulating idiopathic inflammatory proctitis.

A key diagnostic dilemma in such cases is the overlap between intestinal TB and inflammatory bowel disease (IBD), particularly Crohn's disease. Both conditions may present with chronic diarrhea, rectal bleeding, and nonspecific endoscopic findings. However, histological features remain critical in differentiation. Histologically, intestinal tuberculosis is more likely to demonstrate large, confluent epithelioid granulomas with or without caseation and Langhans giant cells, whereas Crohn's disease more commonly exhibits smaller, discrete noncaseating granulomas, basal plasmacytosis, ulcer with fissures, crypt branching, crypt abscess, and crypt distortion.^7^ In this case, there were confluent granulomas with Langhans giant cells. Endoscopically, there were no aphthous ulcers, deep longitudinal ulcers, cobblestoning, skip lesions, or ileocecal involvement. Furthermore, complete clinical and endoscopic resolution following anti-tubercular therapy without immunosuppressive treatment made Crohn's disease unlikely. Other differentials of granulomatous proctitis were less likely. Sarcoidosis is rare in the gastrointestinal tract and is usually associated with multisystem disease, which was absent in our patient. Yersinia infection predominantly involves the terminal ileum and mesenteric lymph nodes rather than the isolated rectum, and the patient lacked an acute infective illness. Amoebiasis typically demonstrates flask-shaped ulcers with trophozoites on histology, neither of which was identified.^8^ Bechet disease is generally associated with recurrent oral or genital ulcers, and characteristic deep punched-out intestinal ulcers were absent in this case.^9^ Sexually transmitted infectious proctitis was considered unlikely because of the patient's age, absence of relevant risk factors, and lack of ulcerative or exudative lesions.

Microbiological confirmation of intestinal TB remains challenging. Nucleic acid amplification tests, including GeneXpert *Mycobacterium tuberculosis*/resistance to the antibiotic rifampicin, as well as Ziehl–Neelsen staining and culture, often have limited sensitivity due to the paucibacillary nature of mucosal disease. Reported diagnostic yields are generally low, particularly in biopsy specimens, and a negative microbiological result does not exclude the diagnosis when histopathology is strongly suggestive.^7^ In our case, both acid fast bacilli staining and GeneXpert testing were negative; therefore, the diagnosis was considered probable isolated rectal tuberculosis, based on characteristic histopathological findings and clinicopathological correlation rather than microbiological confirmation.

A review of the published literature (Table 1) demonstrates that rectal TB predominantly affects adults, with pediatric and adolescent cases being distinctly uncommon. Most reported patients presented with advanced disease characterized by ulcerative lesions, strictures, or rectal mass lesions, frequently accompanied by constitutional symptoms such as fever and weight loss. Hematochezia, constipation, tenesmus, and abdominal pain were the commonest presenting complaints, whereas isolated mild chronic proctitis with increased stool frequency and mucus discharge has rarely been described. Although the majority of reported cases occurred in immunocompetent individuals, rectal TB has also been reported in patients with HIV infection, posttransplant immunosuppression, anti-tumor necrosis factor (TNF) therapy, and even in association with rectal carcinoma, underscoring its diverse clinical settings.^3,12,13,15^ Another recurring theme is its ability to mimic other disorders, including IBD, colorectal carcinoma, dysentery, and ulcerative colitis, often delaying diagnosis.^2,4,10,11,14^ GeneXpert *M. tuberculosis*/mycobacterial growth inhibitor tube positivity has not been reported in any of the cases, reflecting the paucibacillary nature of rectal TB.

**Table 1.** Epidemiologic characteristics in patients with rectal TB

Author	No of patients	Age group	Clinical presentation	Comorbidity	Endoscopic phenotype	Notable feature
Davis^1^	1	Adult	Tenesmus, fever, weight loss	No	Ulcerative lesion	Early classical description of rectal TB
Gupta and Dube^2^	1	Adult	Pain abdomen, tenesmus, fever, weight loss	No	Ulcerative lesion	Emphasized diagnostic confusion with IBD
Rai et al^10^	1	Adult	Pain abdomen, tenesmus, fever, weight loss	No	Ulcerative lesion	Rectal and constitutional symptoms
Puri et al^4^	8	Adults	Hematochezia, constitutional symptoms, constipation	No	Ulceration and strictures	Largest series of rectal TB; commonly having systemic symptoms
Das et al^11^	1	Child	Constipation, intermittent diarrhea, weight loss	No	Stricture	Pediatric rectal stricture presentation
Rege et al^12^	1	Adult	Hematochezia, constipation	No	Mass like lesion	Mimicking malignancy
Sahoo et al^13^	1	Adult	Hematochezia, mucoid stool	No	Ulcerative proctitis	Rectal TB mimicking ulcerative colitis
Sebastian et al^14^	1	Adult	Low backache, hematochezia, weakness, weight loss, fever	Serology reactive for HIV	Mass lesion	Rectal involvement in as case of disseminated TB
Khanya et al^3^	1	Adult	Hematochezia, weight loss, altered bowel habits	Carcinoma rectum	Mass lesion	Coexistence of rectal carcinoma with TB
Patil et al^15^	1	Adult	Constipation, weight loss, rectal prolapse	No	Ulcerative lesion	Rare presentation as rectal prolapse
Santra et al^16^	1	Adult	Hematochezia, fever, diarrhea	No	Ulcerative lesion	Mimicking dysentery
Singh et al^17^	1	Adult	Diarrhea	IBD patient on anti-TNF	Ulcerative proctitis	Overlap of TB and IBD; probably anti-TNF-related reactivation
Liu et al^18^	1	Adult	Pain abdomen, bloody purulent stool, weight loss	No	Ulcerative proctitis and ileitis	Rectal and small bowel TB, coexisting with systemic symptoms
Pandit et al^19^	1	Child	Pain abdomen, weight loss, vomiting	No	Mass lesion	Rare presentation as a mass in pediatric age
Present case	1	Adolescent	Increased stool frequency, mucus discharge, and occasional bleeding per rectum	No	Patchy loss of vascular pattern with erosions	Isolated rectal TB with subtle endoscopic findings

IBD, inflammatory bowel disease; TB, tuberculosis; TNF, tumor necrosis factor

Our patient differed from previously reported cases in several important respects. He was an immunocompetent adolescent who presented without constitutional symptoms, and colonoscopy demonstrated only subtle patchy loss of vascular pattern with superficial erosions rather than the ulcerative, stricturing, or mass-forming lesions typically reported. Cross-sectional imaging was unremarkable, and microbiological investigations, including Ziehl–Neelsen staining and GeneXpert, were negative, making the diagnosis dependent on characteristic histopathological findings and clinicopathological correlation. This case therefore broadens the recognized clinical spectrum of probable rectal TB by demonstrating that the disease may present as isolated mild chronic proctitis with minimal endoscopic abnormalities.

The marked clinical response following initiation of anti-tubercular therapy provided supportive, albeit indirect, evidence for the diagnosis. Notably, CT enterography did not reveal any bowel wall thickening, lymphadenopathy, or other radiological abnormalities, suggesting the absence of transmural or advanced disease. This highlights that early or mucosal-predominant gastrointestinal TB may not be detected on cross-sectional imaging, and that a normal CT scan does not exclude the diagnosis in the appropriate clinical context.

The principal limitation of this report is the absence of microbiological confirmation by culture or polymerase chain reaction; therefore, the diagnosis remains probable rather than definitive. Nevertheless, the characteristic histopathological findings, exclusion of alternative diagnoses, clinicopathological correlation, and complete clinical and endoscopic response with mucosal healing following anti-tubercular therapy collectively support the diagnosis of probable isolated rectal TB.

This case depicts an uncommon presentation of probable rectal TB as isolated mild chronic proctitis with subtle endoscopic abnormalities in an immunocompetent adolescent. It conveys 3 key clinical messages: rectal TB may present as isolated mild proctitis without systemic symptoms; negative microbiological tests should be interpreted in conjunction with histopathological and clinical findings rather than being used to exclude the diagnosis; and early recognition in endemic settings is essential to avoid misdiagnosis as IBD and prevent delays in appropriate therapy.

## DISCLOSURES

Author contributions: G. Bhaduri wrote the manuscript draft, diagnosed and treated the patient. B. Basak made the histopathological diagnosis. AA Ahmed made modifications in the draft and guided the final draft. B. Bandopadhyay provided the colonoscopy images. G. Bhaduri is the article guarantor.

Financial disclosure: None to report.

Informed consent was obtained for this case report.

## ABBREVIATIONS:

CT: computed tomography
HIV: human immunodeficiency virus
IBD: inflammatory bowel disease
TB: tuberculosis
TNF: tumor necrosis factor
